# Supplementary material for: Ab Initio Modelling of the Structure of ToxA-like and MAX Fungal Effector Proteins
Source: Int J Mol Sci. 2023 Mar 26;24(7):6262. doi: 10.3390/ijms24076262 (PMC10094246; doi:10.3390/ijms24076262)

Ab initio modelling of the structure of ToxA-like and MAX fungal effector proteins

Lina Rozano, Yvonne M. Mukuka, James K. Hane and Ricardo L. Mancera

Supplementary Materials

Table S1. Uniprot ID of templates used for ToxA and MAX effectors.

Template PDB ID	Uniprot ID	Reference
Tox A		
1ZLD	P78737 (P78737_9PLEO)	Sarma, Manning, Ciuffetti, & Karplus, 2005
5OD4	A0A0C4DI32 (A0A0C4DI32_FUSO4)	Di et al., 2017
2QVT	Q1HBK6 (Q1HBK6_MELLI)	Wang et al., 2007
MAX		
2MM0	Q9C173 (Q9C173_9PLEO)	Nyarko et al., 2014
5Z1V	A0A0H4ITX1 (A0A0H4ITX1_MAGOR)	Zhang et al., 2018
6FUB	C4B8C2 (C4B8C2_MAGOR)	De la Concepcion et al., 2018
5ZNG	FTJ0N2 (RGA5R_ORYSJ)	Guo et al., 2018
2N37	B9WZW9 (B9WZW9_MAGOR)	Ose et al., 2015
2LW6	H2DQR0 (H2DQR0_MAGOR)	Zhang et al., 2013

Table S2. FASTA sequences of ToxA and MAX effector templates used in Rosetta *ab initio* modelling.

Effector (PDB ID)	FASTA sequence

ToxA (1ZLD)	>ToxA (PDB ID: 1ZLD)<br>APTPEADPGYEIVKLFEAANSSELDARGLSLDWTLKPRGLLQERQGSC<br>MSITINPSRPSVNNIGQVDIDSVILGRPGAIGSWELNNFITIGLNRVNAD<br>TVRVNIRNTGRTNRLIITQWDNTVTRGDVYELFGDYALIQGRGSFCLN<br>IRSDTGRENWRMQLEN
Avr2 (*Fol*) (5OD4)	>Avr2 (PDB ID: 5OD4)<br>MSMTWVCSIAGLPVEDADSSVGQLQGRGNPYCVFPGRRTSSTSFTTSF<br>STEPLGYARMLHRDPPYERAGNSGLNHRIYERSRVGGLRTVIDVAPPD<br>GHQAIANYEIEVRRIPVATPNAAGDCFHTARLSTGSRGPATISWDADA<br>SYTYYLTISED
AvrL567 (2QVT)	>AvrL567 (PDB ID: 2QVT)<br>MEDVPAELTGVSEGYTRFYRSPTASVTLSGLVNVKWDNEQMTMPLF<br>KWIGGEQAEELHFCVHIAHSIGPKLNLARTLGTVNSNMDQHWAQAH<br>RHSGATRRTIQDFHLFANDIPNFPDYIKIKLVPKT
ToxB (2MM0)	>ToxB (PDB ID: 2MM0)<br>NCVANILNINEAVIATGCVPAGGELRIFVGSSHSYLIKATSSCGLSLTN<br>QVFINGESVQSGGR
AvrPib (5Z1V)	>AvrPib (PDB ID: 5Z1V)<br>TQVTILKKGERITWVEVPKGESREFNIRGKYFTVSVSDDGTPSISGSKY<br>TVE
AvrPik (6FUB)	>AvrPik (PDB ID: 6FUB)<br>ETGNKYIEKRAIDLSRERDPNFFDNPGIPVPECFWFMFKNNVRQDAGT<br>CYSSWKMDMKVGPNWVHIKSDDNCNLSGDFPPGWIVLGKKRPGF
Avr1CO39 (5ZNG)	>Avr1CO39 (PDB ID: 5ZNG)<br>WKDCIIQRYKDGDVNNIYTANRNEEITIEEYKVFVNEACHPYPVILPD<br>RSVLSGDFTSAYADDDESC
AvrPia (2N37)	>AvrPia (PDB ID: 2N37)<br>APARFCVYYDGHLPATRVLLMYVRIGTTATITARGHEFEVEAKDQNC<br>KVILTNGKQAPDWLAAEPY
AvrPiz-t (2LW6)	>AvrPiz-t (PDB ID: 2LW6)<br>SFVQCNHHLLYNGRHWGTIRKKAGWAVRFYEEKPGQPKRLVAICKN<br>ASPVHCNYLKCTNLAAGFSAGTSTDVLSSGTVGSIGNDPQAQRQ

Table S3. UniProt and GenBank identifiers of structurally-unconfirmed effectors used in Rosetta *ab initio* modelling.

Effector	Gene name	UniProt ID	GenBank ID	Fungal
Avr2	Avr2	A0A0A7HTX0 (A0A0A7HTX0_PASFU)	AIZ11403.1	*Fulvia fulva*
AVR4E	AVR4E protein	Q5VB04 (Q5VB04_PASFU)	AAT28197.1	*Fulvia fulva*
Avr5	AVR5	A0A023UJQ9 (A0A023UJQ9_PASFU)	AHY02126.1	*Fulvia fulva*
AVR9	Race-specific elicitor A9	P22287 (AVR9_PASFU)	P22287.1	*Fulvia fulva*

AVRA1	CSEP0008 putative effector protein	N1JGD1 (N1JGD1_BLUG1)	CCU81904.1	*Blumeria graminis f. sp. hordei*
AvrL2-A	AvrL2-A	A0A1B2CW13 (A0A1B2CW13_MELLI)	ANY58853.1	*Melampsora lini*
avrLm1	avrLm1 protein	Q258K5 (Q258K5_LEPMC)	CAJ29326.1	*Leptosphaeria maculans*
AvrLm11	AvrLm11 protein	E4ZT77 (E4ZT77_LEPMJ)	XP_003838051.1	*Leptosphaeria maculans*
AvrLm6	AvrLm6 protein	A4F4L2 (A4F4L2_LEPMC)	CAJ90695.1	*Leptosphaeria maculans*
AvrPi9	Zn2-C6 fungal-type domain containing protein	G4NJP7 (G4NJP7_MAGO7)	XP_003721206.1	*Magnaporthe oryzae*
AVR-Pii	AVR-Pii protein	C4B8B7 (C4B8B7_MAGOR)	ELQ63308.1	*Magnaporthe oryzae*
AvrStb6	AvrStb6	A0A2K9YVY7 (A0A2K9YVY7_ZYMTR)	AUW40142.1	*Zymoseptoria tritici*
BAS1	Biotrophy-associated secreted protein 1	G5EHI7 (BAS1_MAGO7)	XP_003710761.1	*Magnaporthe oryzae*
BAS162	MGG_09379	G4NI07 (G4NI07_MAGO7)	XP_003720234.1	*Magnaporthe oryzae*
BAS2	Biotrophy-associated secreted protein 2	G5EI08 (BAS2_MAGO7)	XP_003717663.1	*Magnaporthe oryzae*
BAS4	Biotrophy-associated secreted protein 4	G5EI20 (BAS4_MAGO7)	XP_003717679.1	*Magnaporthe oryzae*
CgEP1	Host-nuclear effector	E3QDG3 (E3QDG3_COLGM)	XP_008092955.1	*Colletotrichum graminicola*
ECP1	ECP1	Q00364 (Q00364_PASFU)	QDX18247.1	*Fulvia fulva*
ECP2	ECP2 protein	Q00365 (Q00365_PASFU)	QDX18258.1	*Fulvia fulva*
ECP4	ECP4 protein	Q9P408 (Q9P408_PASFU)	ABM47072.1	*Fulvia fulva*
ECP5	ECP5	Q9P407 (Q9P407_PASFU)	ABM47073.1	*Fulvia fulva*
eff1-1	Effector family protein Eff1-1	Q4PDL7 (Q4PDL7_USTMA)	XP_011387751.1	*Ustilago maydis*
Iug6	Mo_GLEAN_10000617	A0A0A7DLN4 (A0A0A7DLN4_MAGOR)	AIP86908.1	*Magnaporthe oryzae*

MiSSP7	MISSP7.2	B0DD66 (B0DD66_LACBS)	XP_001881946.1	*Laccaria bicolor*
MoCDIP4	CBM1 domain-containing protein	A0A4P7NBE8 (A0A4P7NBE8_MAGOR)	QBZ57550.1	*Magnaporthe oryzae*
MoHEG13	MGG_09378	G4NI08 (G4NI08_MAGO7)	XP_003720235.1	*Magnaporthe oryzae*
NIP2	NIP2 avirulence protein	U3NHN5 (U3NHN5_9HELO)	AGW29229.1	*Rhynchosporium commune*
NIP3	NIP3 avirulence protein	I1VGT8 (I1VGT8_9HELO)	AFI43936.1	*Rhynchosporium commune*
pep1	UMAG_01987	A0A0D1E411 (A0A0D1E411_USTMA)	XP_011387901.1	*Ustilago maydis*
Pit2	Secreted effector Pit2	A0A0D1EAR7 (PIT2_USTMA)	XP_011387264.1	*Ustilago maydis*
PpEC23	CSEP-35	A0A142KWI6 (A0A142KWI6_PHAPC)	AMS24306.1	*Phakospora pachyrhizi*
PstPEC6	Kazal-like domain containing protein	A0A2S4WEB9 (A0A2S4WEB9_9BASI)	POW20114.1	*Puccinia striiformis*
PstSCR1	PSTT_01941	A0A2S4W1V6 (A0A2S4W1V6_9BASI)	POW15657.1	*Puccinia striiformis*
RTP1	Rust transferred protein	Q334H6 (Q334H6_UROFA)	CAI96535.1	*Uromyces fabae*
SPD10	MGG_11991	G4MPQ6 (G4MPQ6_MAGO7)	XP_003709017.1	*Magnaporthe oryzae*
SPD2	MGG_12942	G4N480 (G4N480_MAGO7)	XP_003712607.1	*Magnaporthe oryzae*
SPD4	MGG_14422	A4UBM2 (A4UBM2_MAGO7)	XP_003711101.1	*Magnaporthe oryzae*
SsSSVP1	CVNH domain-containing protein	A7E9T8 (A7E9T8_SCLS1)	XP_001597872.1	*Sclerotinia sclerotiorum*

Table S4. FASTA sequences of structurally-unconfirmed effectors used in Rosetta *ab initio* modelling.

Effector	FASTA sequence
Avr2 (*F. fulva*)	>Avr2<br>KKLPGCDKDPCKVKEKSGKYKLKIGAKCSATCDGKLTRGGTCENVQGNHLCCFGLCG
AVR4E	>AVR4E<br>DFSRDCPPGSGVGNQAEWSARGVDGTAIPRELDAHSLCDCFKPFLNVLGCSVTSVVTERAVFVKGHLNYCARMSESVAGISPCKEWEIEVGGAHPERMANR
Avr5	>Avr5

	SYDALPINCRDTTNYCFNGNGRHEVCSYCNQAKEEPLKLGRRGGQRDCG<br>VAGSQCNDVDHQQCDARCCSKIGSPTFYGVRCPYPY
AVR9	>AVR9<br>AALPVGLGVGLDYCNSSCTRAFDCLGQCGRCDFHKLQCVH
AVRA1	>AVRa1<br>RTWQCPSGDILYEEDVYSHTNMNSFEHFPFKTIEDEVYENTSLSRGVVYK<br>VLDVHPSSSSATTFAVFTSNKRVAVVYQQGYRNNRKFIEECT
AvrL2-A	>AVR2a<br>KKLPGCDKDPCKVKEKSGKYKLKIGAKCSATCDGKLTRGGTCENVQGN<br>HLCCFGLCG
avrLm1	>avrLm1<br>SPATKNNVNQPLDNISRRSEWKSVQISPVKEHSAKTADNTENNHNLEKR<br>VFTSPHMKRTFTLALENTFYAMAWLIDFSFSDDGEPHFSYKLQSFNHEDN<br>PPKILADVVVPLITTSYNSANQYRGKANVELLCHLAKEYVHVYFSVEVFA<br>SGASFVIGKIIDYPTVYVNNQFRKVVKFDIAGAI
AvrLm11	>AvrLm11<br>FEGRPCCQKWIEQCKLEGAVGCMLADYNYCVVPSSTCQEQCNGFFGRN<br>GRSTHGRDTEYVYQIHNMYTANEKDPTC
AvrLm6	>AvrLm6<br>QPHLLCACESGRRDGVDDTRTLKVVKGTGGRFVFSSRYWTKAEGAPHE<br>GNYAHAINGTITKKGTNIQAHDDGLIGGEEMNSLCPEHSTCFSPNLKAKS<br>THSCGPDGKYGCVSAWLSVNWEGQIQ
AvrPi9	>AvrPi9<br>LPAGGLPGSPGSAVQRCHCPPRGSHAHGSLAAREEAPEAEGDAKISARYT<br>CPNCHKTGKGCDDGWCQVEKTHW
AVR-Pii	>AVR-Pii<br>LPTPASLNGNTEVATISDVKLEARSDTTYHKCSKCGYGSDDSDAYFNHK<br>CN
AvrStb6	>AvrStb6<br>RVSCGGIGDLCKAGDSCCNYPGTDCFQDGQYPRCHTACGHFQFGFCHDG<br>KQCNCQVILGCGCV
BAS1	>BAS1<br>ADQGSNTFDQRYQGYPWRPANGPIREEKQENVGHRRGGAEYFTSGSPSI<br>AAEDYSAKNAPSRFEQWKAQQKERAERKQDRGLNGIRRVENYYP
BAS162	>BAS162<br>GGGTYIPKDPALAHRAPEKFPWSGLLNNPFMTKPVIWYQIGQIKIWTDTR<br>DVTYVRDGTKFDTYDQCLSHCLKYSVFNKSKARSSGSQGQTSEYRGRR<br>MHH
BAS2	>BAS2<br>NVTPNDAGAKNVGTGNGQQFITGGCVNGTDCQSRCCAGNGENKGVCSN<br>EVAANQNGKTGCGFEDPNKAQTVKEAKEQVKKQGF
BAS4	>BAS4<br>DSHQNLVCVRTYPDNNSAENLEATRCACDWLKKNGKCDDCTIWENRLC<br>HSDAKSLDGNEFEDACVRKCPNLGATGSSIPPA
CgEP1	>CgEP1

	APTAPINDVAAPVEARAADFVDGAHLEARKNKKKKKEGKKDKKAKDN<br>ARDLTELEDRDADDVTIVEIRTPEFVADDAHNLEARKNKKKKKKGGKK<br>DKKEKKAKDNARDLTELEARDEEADDAAQLEARKNKKKKKGGKKDKK<br>EKKAKNNARDLTELEARDEEAEDAAQLEARKNKKKKKGGKKDKKEKK<br>AKNNARDLTELEARDEEADDAAQLEARKNKKKKKGGKKDKKEKKAKN<br>NARDLTELEARDEEADDAAQLEARKNKKKKKGGKKDKKEKKAKNNAR<br>DLTELEARDEEAEDAAQLEARKNKKKKKGGKKDKKEKKAKDAGAA
ECP1	>ECP1<br>TVQGGAPVDDLKFAKKFNQNCQQISGGPNGAICPDGDLYWCKDGRAIFC<br>QTCQTGCTADENGKVGYCNEGPTNPKCL
ECP2	>ECP2<br>RNAGNSPGSNRCDASTFNNGQDFDIPQAPVNDCRQMVENINRDSQFSVS<br>HSWARPFGGYGDCAFNVRVIAGWRNGLVGGADAVDLLTDSVKNFGEA<br>NKVSSKGTYNQIVSAEGEVTCDSVDRGGQVRVQWIVASSSYNPSNDD
ECP4	>ECP4<br>DPSFRFSEYGNGDTTCSPDNLISPSVSIDDGFGGCKQFPANTNSVQYALLE<br>GSRAGCIVKLFTNNACNESADSEHVPAVGETKCQPPIGGGWQSVQVTCP
ECP5	>ECP5<br>RGDNKPGQFNYICEDIPCDEEGVDSWILQACIGIGGTELTAHGGIYDTTGE<br>LVGRTAICLCARGQTKAHDYNISGDYPPGVAHLTFDVRAPYWCQSSG
eff1-1	>eff1-1<br>WPYPSGNHPDNADGARRGEQSPLPGFSVAHPSSPQNEFDPDLLRSLLAKI<br>ESEASSHPHVAFQGDGHYYPNVNQPSSLYQASQHGFGSLSRDKGQVGSS<br>QARESFSDTQGLSSSAEPAFKYRKTVQRVADALRQFQANNRLEGAQALR<br>DIIQRHPQLDTTSGSQSQGFRNNFGAAPSVPSRESQGGTIHHGPGDTKVV<br>DIPSDRIEAQTTAEESHESQPLAGHGDGRYRSLPASRADELRKIPTTMLAL<br>PPPSRTRYIYDQVDDPAIRDNINNQVFAGKLVWIDRARIPASRIVSSRKQM<br>FRPHRVLPMTNFPEIHLSDGKGSIKDVRFTFHGGGSYRLMSWPEGYNLVE<br>GQNYLAFWGIPEGRQTGRPMLMQNYGYAFLPPQHRLEVNEHLWALKKE<br>IADKASEATWMHA
Iug6	>Iug6<br>NTVAPADVAAVDAPRAPLLVRRNCEGKNTQAECERFKWLTGCTWLRGS<br>KYCSST
MiSSP7	>MISSP7<br>SPVPGEVGLVERGPIPNAVFRRVPEPNFFKDLLRALGQASQGGDLHR
MoCDIP4	>MoCDIP4<br>HYIFSIVFVNGVQKGGDYTYIRKNSNTYMPSFTNDIVNSPDLRCNTGARA<br>NSGTQTLTVKAGDKIGFKLWYNEFIEHPGPGFVYMSKAPGSVASYDGSG<br>DWFKAYETGLCGGQPNSDGAWCTWQKDRIEFTIPPRTPPGEYLVRVEHL<br>AIHESHVGKGQFYMECAQFKVEGPGGGSPGPLVKIPGLYKASDPGIAYN<br>KWTNNPARYVMPGPAVWADNGSSNPSNPPSNPPSNGGGGGGGSGSGAP<br>LYGQCGGEGWTGPKTCAQGTCKVSNQYYSQCL
MoHEG13	>MoHEG13<br>HPLDNGGLEPRQQKICSPNGHTDGDLTNPNSLCTLCNSGCQRVAGQPAC<br>CS

NIP2	>NIP2<br>YYVVVCVPRDGAEIGDVEWAIQNRRHDLALGGKGFWRGHSTSCHRNAN<br>AVVDVVALCRSDPYIGAHPTVLKYGASVLCQASGAPDWPTCTVNC
NIP3	>NIP3<br>ACGDVYTIKGNCTIRDYKCKYPRVPSCNSKGACPYLHLFDVPIFCDQDKD<br>CVTSYKCNKNYKRSPLNLGPFYEDGDLVAKEEGEGGEGDSELVEEDDE
pep1	>pep1<br>DAAGAVPLPNFKVDPQPLASTFYWFSSVEVGVCYNPQARVGSIKGALHC<br>THQENYDRDNNSYTLPQTCVALKPLGKAFSSNVRDSCTNAKGIFNVIVPA<br>SSNALGSQAYDAVQAKGGTGGTGTDDDTSAPDSNDQEKKGGGLLGGIG<br>SMFGM
Pit2	>Pit2<br>IPVRRSLSTDASMSSAAGKLNRRWWFGFTGSLGKEPDNGQVQIKIIPDALI<br>IKNPPANKDDLNKLIENLKRKHPRFKTVVMPTDPNGDVVIWE
PpEC23	>PpEC23<br>ASTSEPSGQTLDCSHYTGANTKEATCNEFPGRICHGGCTGAVVASNCTLN<br>PGEEPKDQTYTIAFGKSSATISICRNEKGSYSCTGPIKGSAKCSVCVDPPTG<br>SDQSPTTPAPAPGNNGSTSKEGQTLQCTHFTGANTQSATCNEVPGRVCN<br>KGCTSSVVATKCTLNPGDQESQQNCTQAFGKSSAAISICINDKGSFSCTGS<br>VSGNATCSGCTDQSSSGSSEYPGSSGTTKPPTDPTGGENKDQDKKAEATS<br>LQFAMSSFCLALSLMIGVAVL
PstPEC6	>PSTPec6<br>SGASTPKKCKKTIMHEKDKCWTIGCTEEVSTAKWRLQCDDKNCAHPVC<br>SGDWTPTKAICFVCLGNR
PstSCR1	>PstSCR1<br>FKCPGLHGTPSQTHGYCTRSITDEERKAKKIGKEFTMWKEEIKTVDGKFS<br>CDKVDLNGSVATDSFCCDVAGRIGEVEKSKQAMWTNNCSKAS
RTP1	>RTP1<br>QLVGSDVVLVRTSHQSMGKASVSYCREMTRQHHKRELDQDANPGHRR<br>HKSEPEGVKPSNHTSAPTPSSPPLTTVDLTPAKLNTACYPGTFQAPLLEDC<br>EVVIRAQLYNSTGSLQVSPGDYVFVSYGTCATVFQNPQYSKYSLQYNWA<br>ELGYVGGKLAGRCLLPEDHSMGGTAVFDTYLGRTYPDVIISLQRFDDRD<br>FIIPE
SPD10	>SPD10<br>QCFDDLDCLFSGIENAKCAKATNTDLLGYETPPPCLLFTPKILGISADLFH<br>NQSASRTCKGPA
SPD2	>SPD2<br>QSCRQLSADFCQCGTYCYRTRDGGCNPAGTFYGLCPLKRETTFEA
SPD4	>SPD4<br>LYTHHLTVDSHCPSLRSAVASRPGRQEVLEHNPCRKSDALVVITKNDESV<br>PP
SsSSVP1	>SsSSVP1<br>APLEPRQSIWGKPTDVSVQYCYNDFPCINAREAWQSCQVSSLPGSYSQIT<br>DPEARNTVVAQCLCDNKIVNETASCISCLSYASGNFLVLDTVEQHAQDM<br>CDKKTTLSQYENYVYQFGGLLQFPLILPSTWLSGTKVNLPDQILIKD

Table S5. Secondary structure characteristics of effector templates according to DSSP and STRIDE.

Effector PDB	DSSP	STRIDE
1ZDL	3% helical (1 helices; 4 residues)<br>49% beta sheet (8 strands; 58 residues)	5% helical (1 helices; 6 residues)<br>54% beta sheet (8 strands; 64 residues)
5OD4	2% helical (1 helices; 3 residues)<br>41% beta sheet (8 strands; 53 residues)	43% beta sheet (10 strands; 56 residues)
2QVT	2% helical (1 helices; 3 residues)<br>28% beta sheet (10 strands; 42 residues)	2% helical (1 helices; 3 residues)<br>30% beta sheet (10 strands; 45 residues)
2MM0	50% beta sheet (6 strands; 32 residues)	59% beta sheet (6 strands; 38 residues)
5Z1V(A)	53% beta sheet (6 strands; 34 residues)	54% beta sheet (8 strands; 35 residues)
5Z1V(D)	53% beta sheet (6 strands; 34 residues)	50% beta sheet (6 strands; 32 residues)
2LW6	47% beta sheet (8 strands; 38 residues)	57% beta sheet (8 strands; 46 residues)
2N37	36% beta sheet (5 strands; 24 residues)	48% beta sheet (7 strands; 32 residues)
5ZNG	44% beta sheet (7 strands; 34 residues)	44% beta sheet (7 strands; 34 residues)
6FUB	3% helical (1 helices; 3 residues)<br>40% beta sheet (6 strands; 38 residues)	3% helical (1 helices; 3 residues)<br>41% beta sheet (6 strands; 39 residues)

Table S6. Structural quality indicators for the best models of ToxA-like, MAX and structurally-unconfirmed effector proteins based on Verify3D, PROCHECK, Ramachandran statistics and PROSA analysis.

Effectors (species)	Verify3D (% residues with 3D-1D score > 0.2)	PROCHECK (error, warning, pass)	Ramachandran statistics (% residues in favourable, allowed and disallowed regions)	PROSA Z-score
Effector templates				
ToxA *(Pyrenophora tritici-repentis)*	61.73	0, 3, 5	85.5, 14.5, 0	-3.70
Avr2 *(Fusarium oxysporum* f. sp. *lycopersici)*	61.29	0, 2, 6	86.7, 13.3, 0	-3.53
AvrL567 (*Melampsora lini)*	77.17	2, 1, 5	84.5, 14.5, 0.9	-4.32

ToxB *(Pyrenophora tritici-repentis)*	43.75	2, 0, 6	79.2, 20.2, 0	-4.01
AvrPib *(Magnaporthe oryzae)*	100.00	0, 0, 8	93.0, 7.0, 0	-5.68
AvrPik *(Magnaporthe oryzae)*	87.10	0, 3, 5	81.3, 18.7, 0	-3.53
Avr1CO39 *(Magnaporthe oryzae)*	31.34	2, 1, 5	76.7, 23.3. 0	-3.28
AvrPia *(Magnaporthe oryzae)*	57.58	0, 4, 4	80.4, 19.6, 0	-4.34
AvrPiz-t *(Magnaporthe oryzae)*	45.56	0, 1, 7	86.7, 13.3, 0	-3.93
Structurally-unconfirmed effectors				
Avr2 *(Fulvia fulva)*	100.00	0, 2, 6	82.2, 17,8, 0	-5.32
AvrStb6 *(Zymoseptoria tritici)*	100.00	1, 1, 6	85.4, 14.6, 0	-4.73
BAS2 *(Magnaporthe oryzae)*	100.00	0, 1, 7	84.6, 15.4, 0	-4.4
MiSSP7 *(Laccaria bicolor)*	0.00	0, 2, 6	90.9, 9.1, 0	-2.8
BAS4 *(Magnaporthe oryzae)*	100.00	0, 2, 6	90.1, 9.9, 0	-3.75
PstSCR1 *(Puccinia striiformis* f. sp. *tritici)*	63.04	0, 1, 7	86.2, 13.8, 0	-1.13
Ecp1 *(Fulvia fulva)*	98.70	0, 1, 7	86.4, 13.6, 0	-4.67
AVRA1 *(Blumeria graminis* f. sp. *hordei)*	58.70	1, 1, 6	89.3, 10.7, 0	-4.85
Iug6 *(Magnaporthe oryzae)*	100.00	0, 1, 7	93.5, 6.5, 0	-5.82
PstPEC6 *(Puccinia striiformis* f. sp. *tritici)*	69.70	0, 1, 7	87.7, 12.3, 0	-5.41

Figure S1. *Ab initio* models (*nstruct* = 50,000) with the lowest RMSD value for each template and their respective native structure using PyMOL Open Source (Schrödinger, LLC) (super function). The Rosetta model is shown in blue and the reference (native) structure in pink.

Effector name/ PDB ID	Rosetta model	Native structure	RMSD (Å)

ToxA (1ZLD)<br>S_00027133	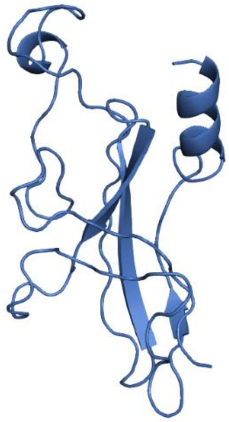	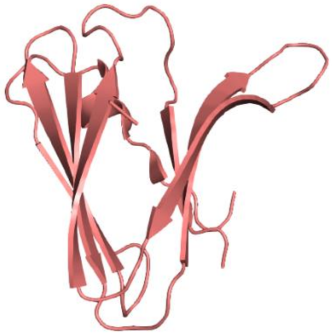	9.642
Avr2 (*Fol*)<br>(5OD4)<br>S_00022933	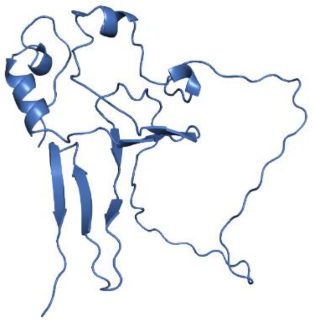	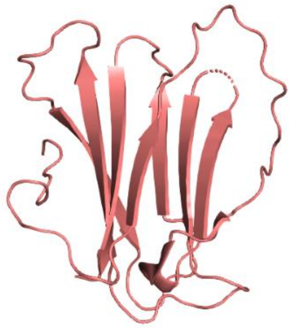	14.925
AvrL567<br>(2QVT)<br>S_00028258	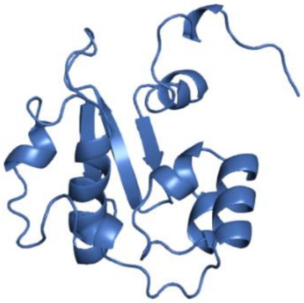	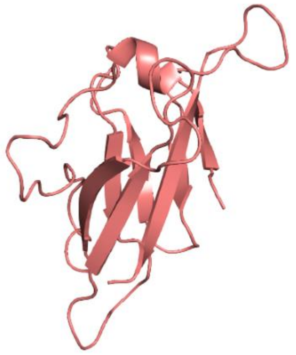	0.482

ToxB (2MM0) S_00048503	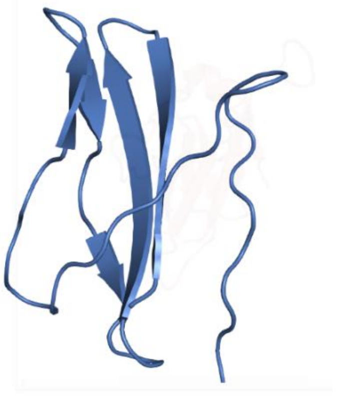	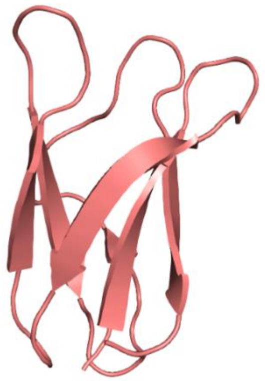	5.003
AvrPib (5Z1V) S_00012083	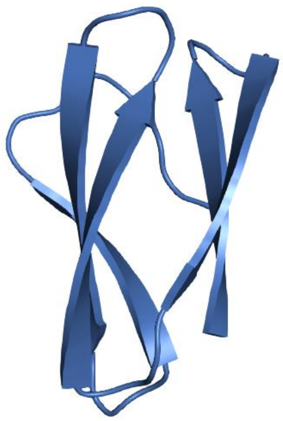	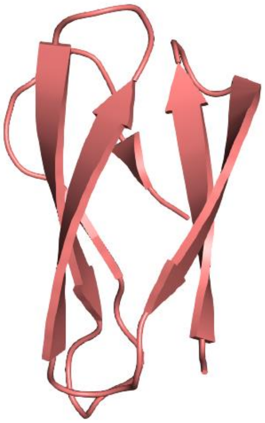	0.732
AvrPik (6FUB) S_00026064	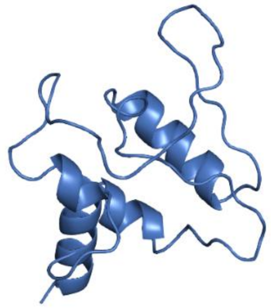	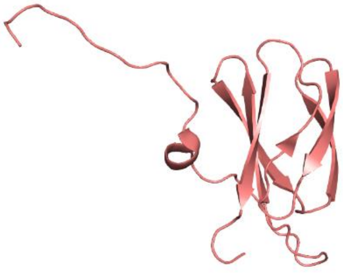	1.765

Avr1CO39 (5ZNG) S_00045958	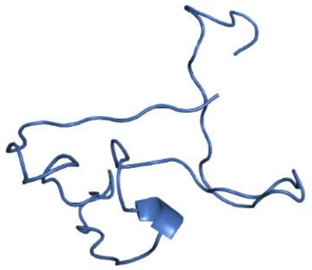	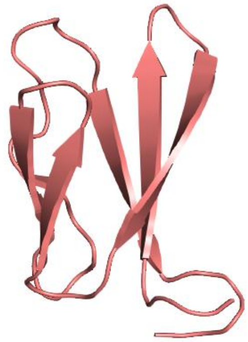	8.546
AvrPia (2N37) S_00047076	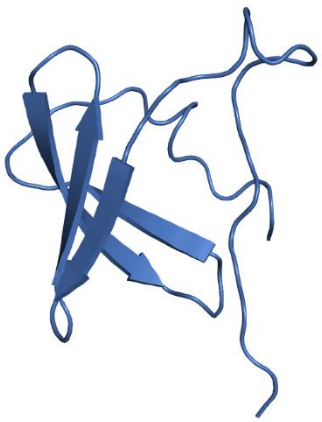	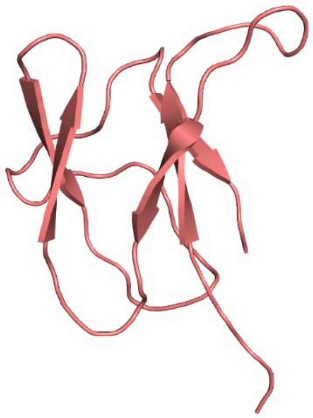	3.380
AvrPiz-t (2LW6) S_00013493	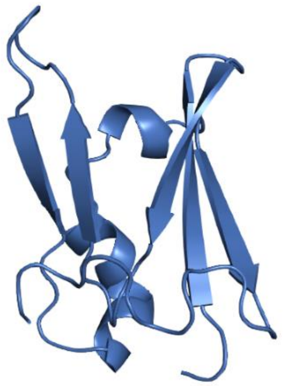	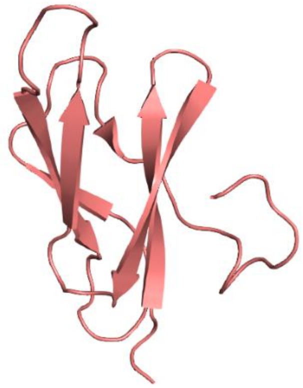	4.113

Figure S2. Structures corresponding to the centroids of the top 10 largest clusters obtained using MaxCluster analysis for each structurally-unconfirmed effectors. The final model (a) for each one of these structurally-unconfirmed effectors is followed by other members (b)-(j). All models are shown with a rainbow colour scheme, from dark blue at the N-terminus to dark red at the C-terminus.

Avr2 (*F. fulva*)

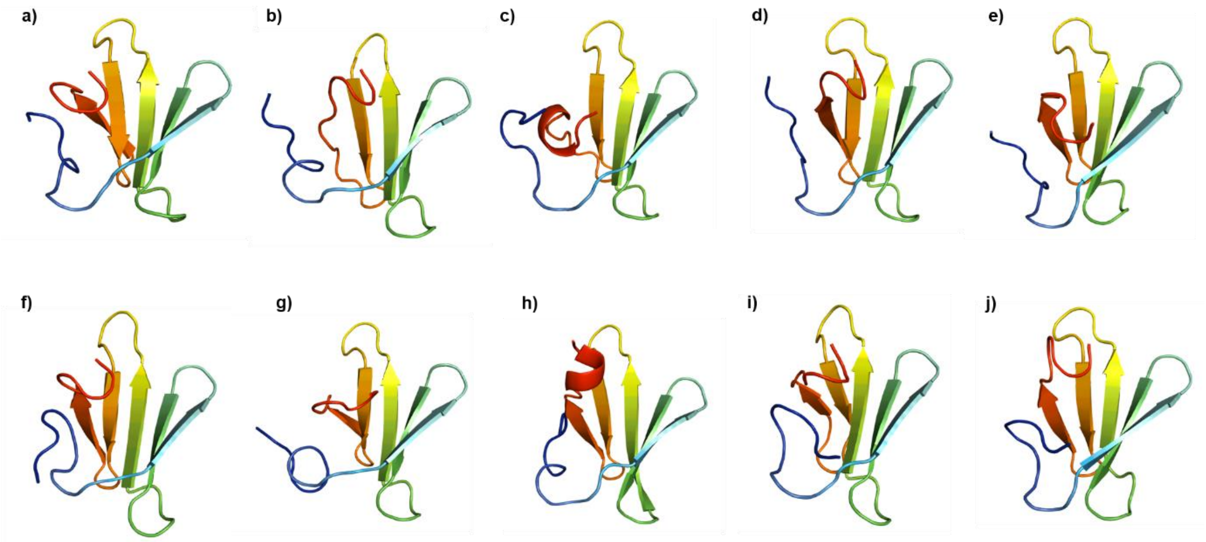

AvrStb6

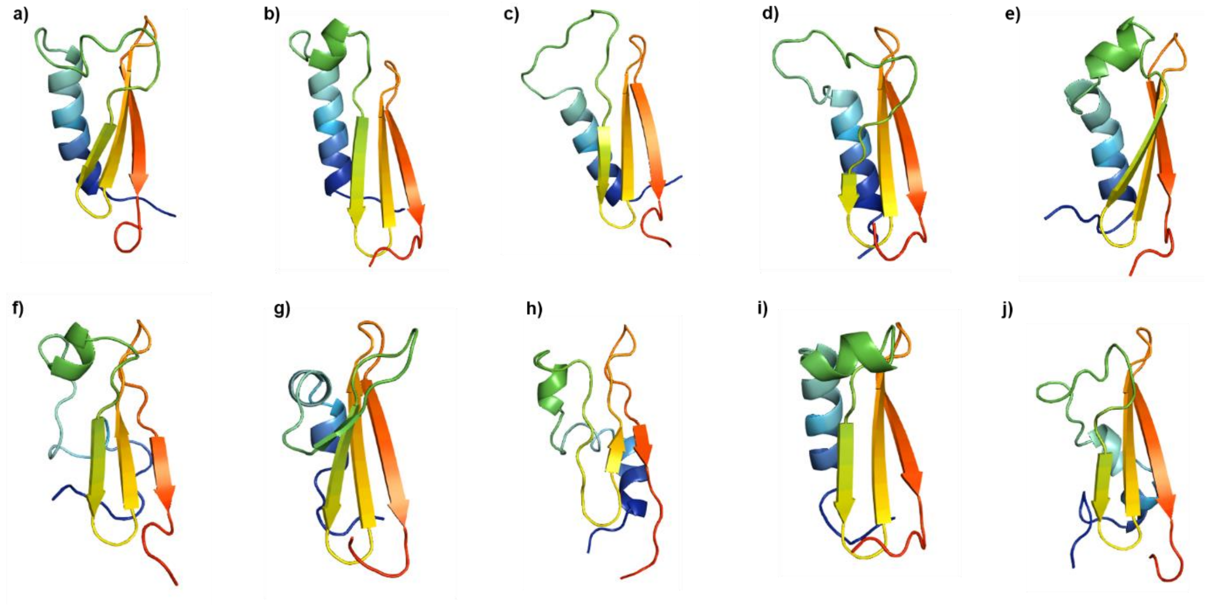

BAS2

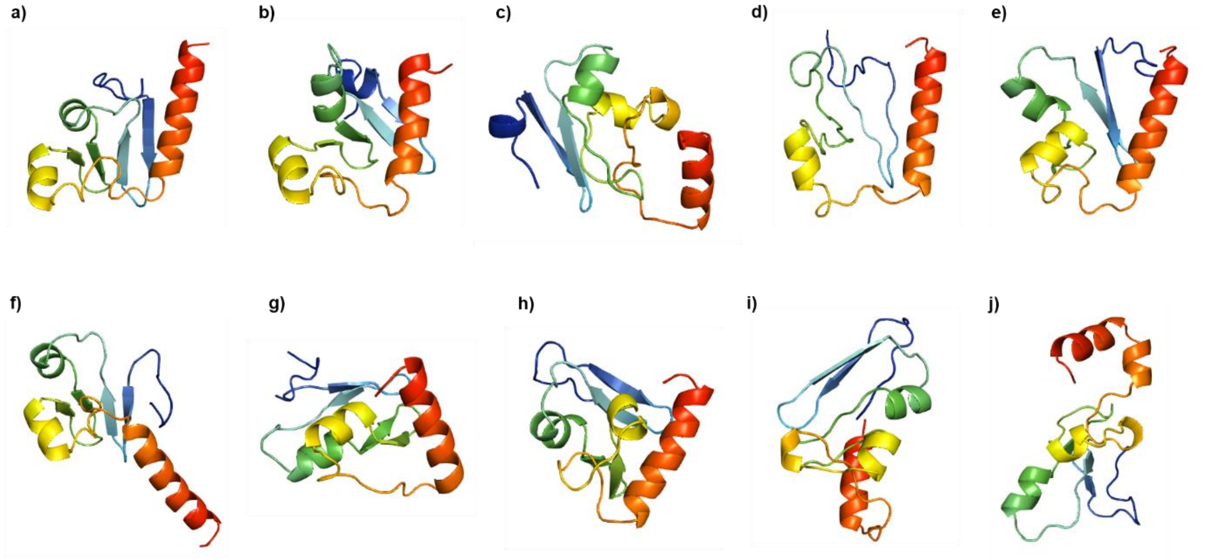

MiSSP7

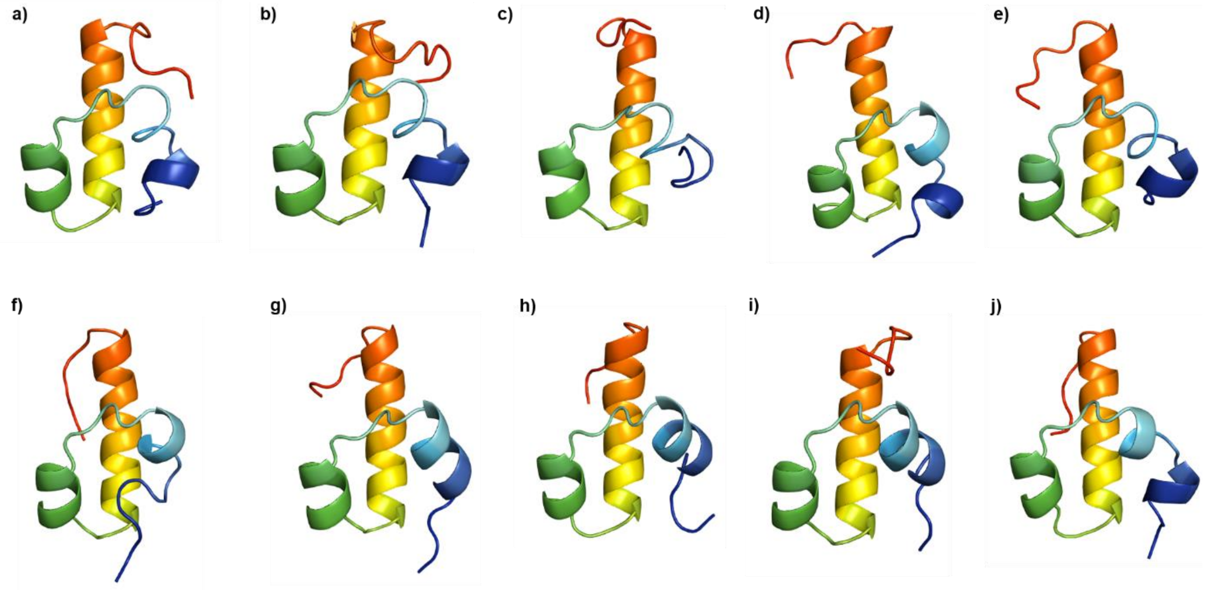

BAS4

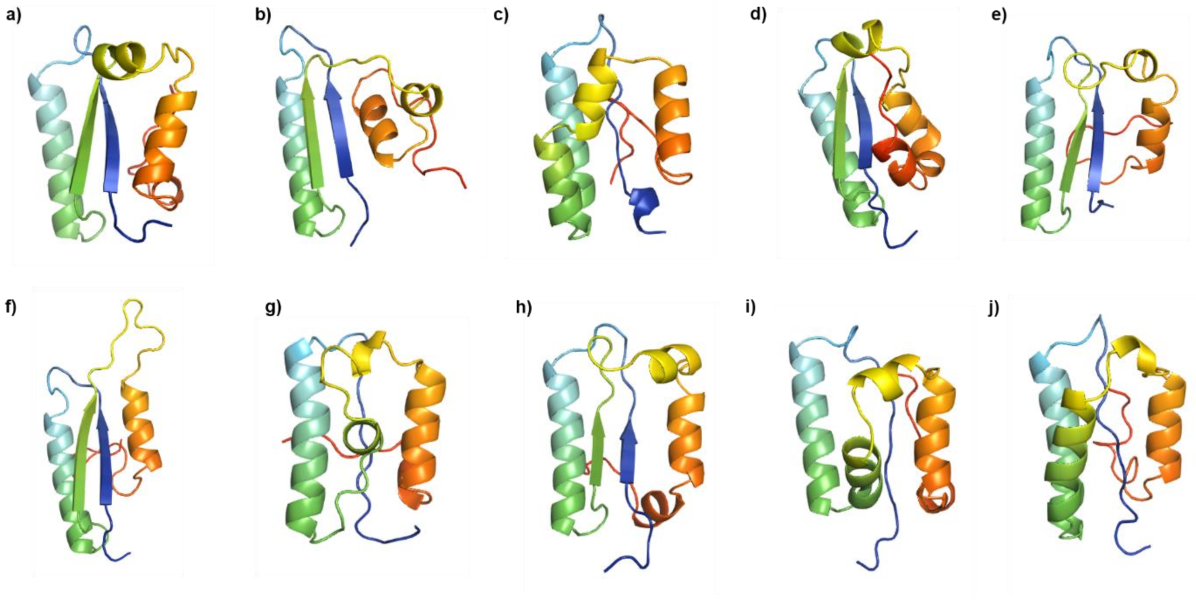

PstSCR1

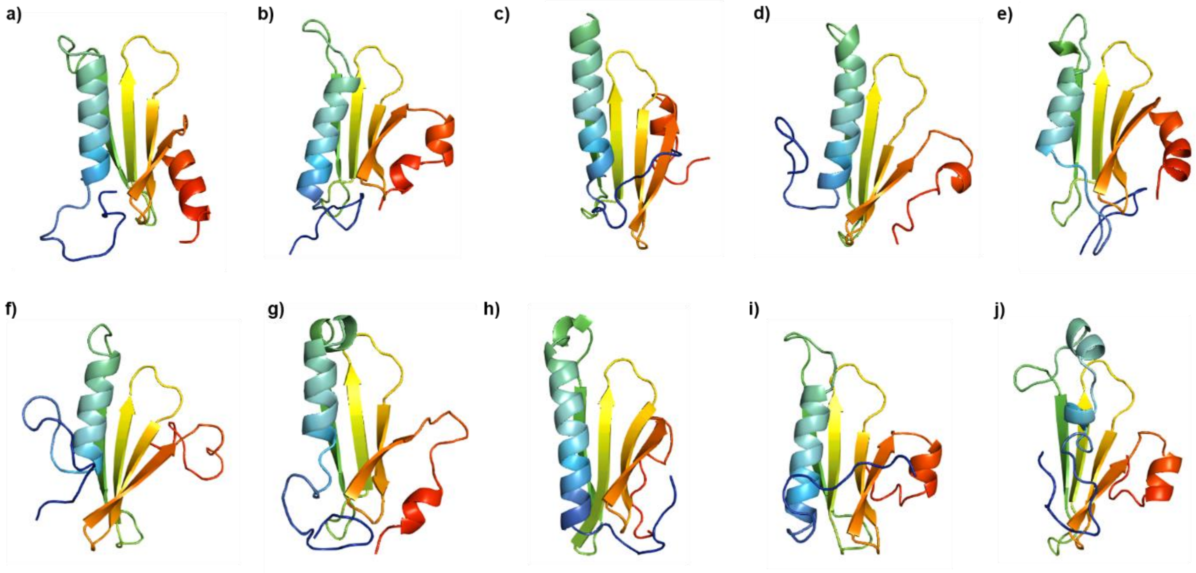

ECP1

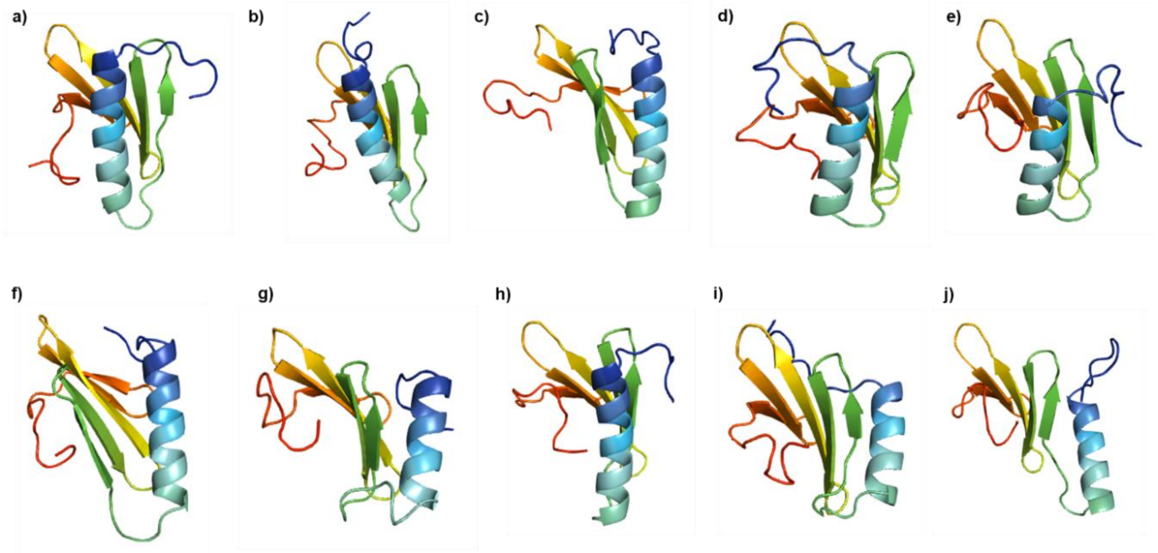

AVRA1

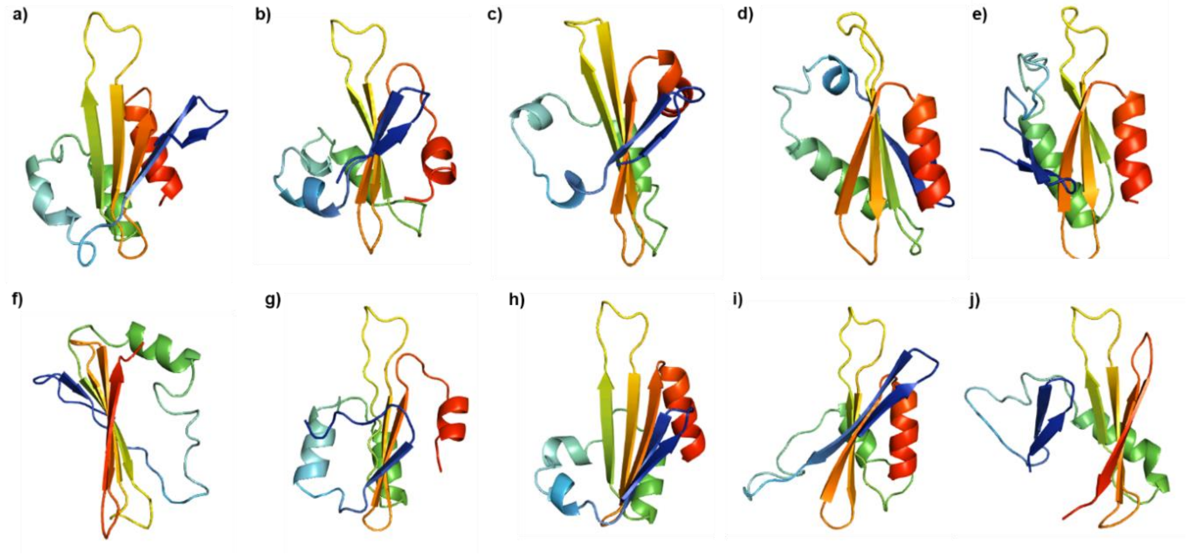

Iug6

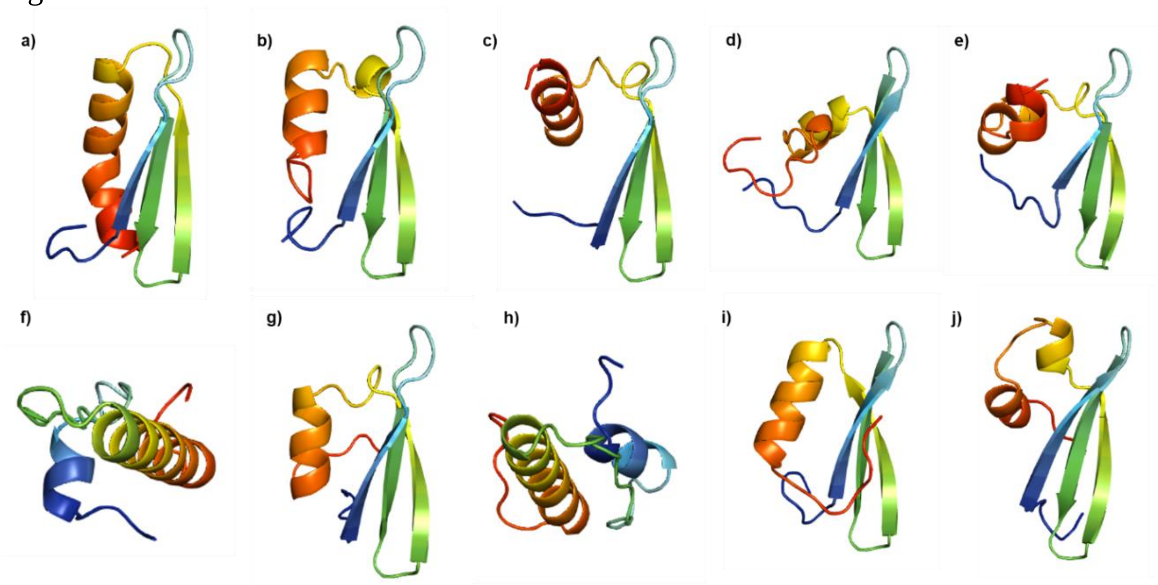

PstPEC6

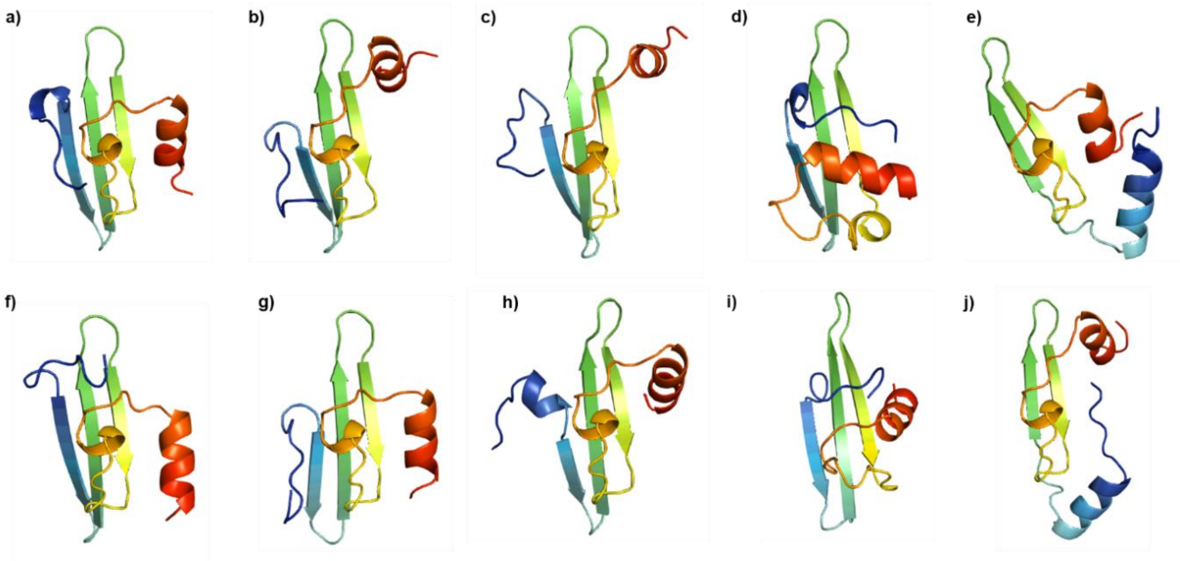

Figure S3. Comparison of *ab initio* models of structurally-unconfirmed effectors obtained with Rosetta (left) and AlphaFold v2.1.0 Colab (right). Protein modelling using AlphaFold was performed with no template in multiple sequence alignment mode and a single model was predicted for each effector candidate. All models are shown with a rainbow colour scheme, from dark blue at the N-terminus to dark red at the C-terminus.

Rosetta models

AlphaFold models

a) Avr2 (*F. fulva*)

b) AvrStb6

c) BAS2

d) MiSSP7

e) BAS4

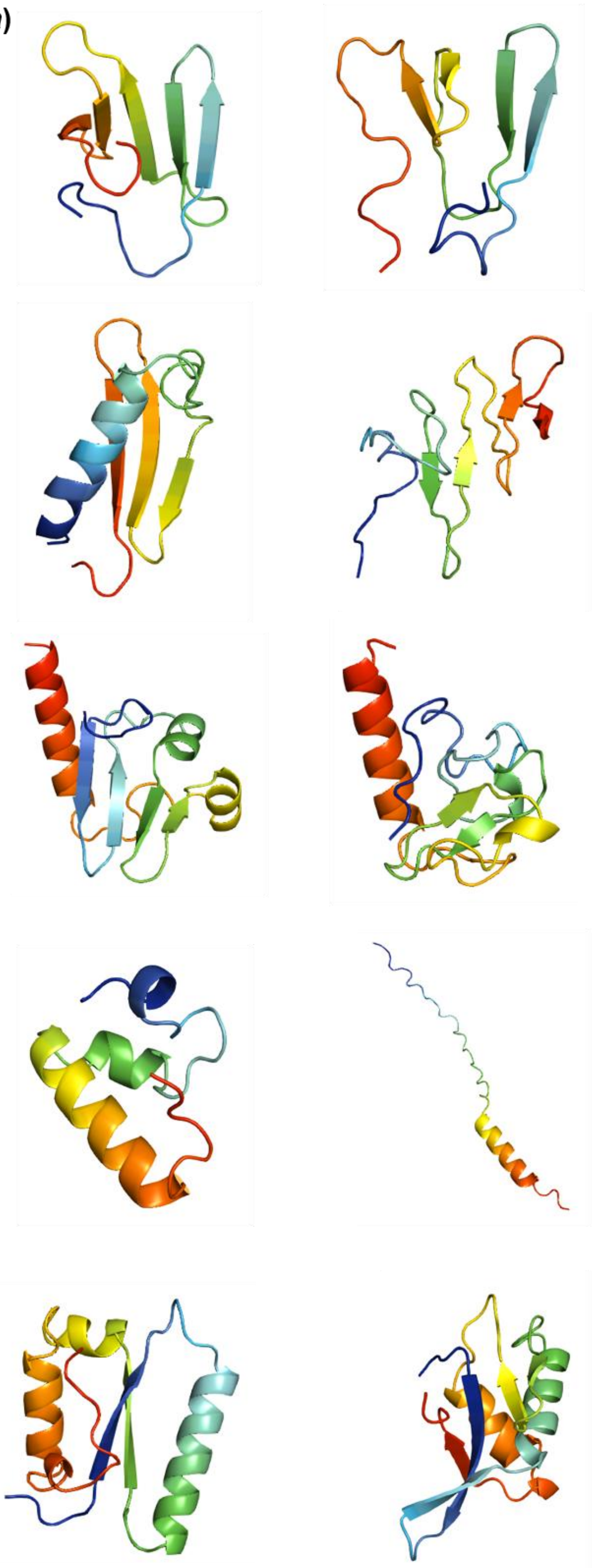

f) PstSCR1

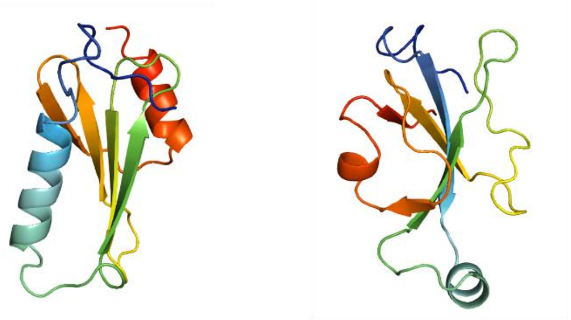

g) ECP1

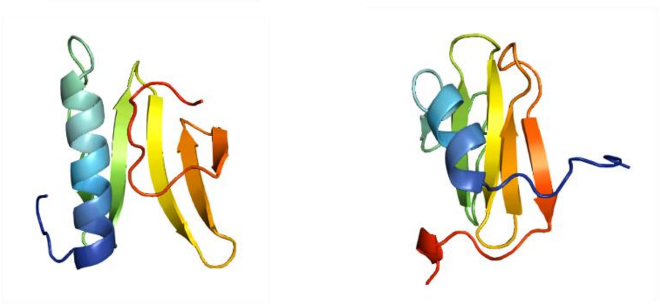

h) AVRA1

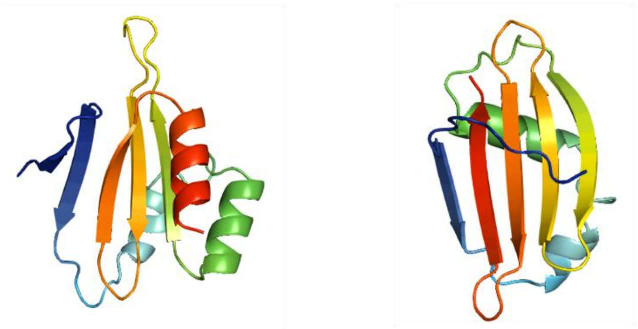

i) Iug6

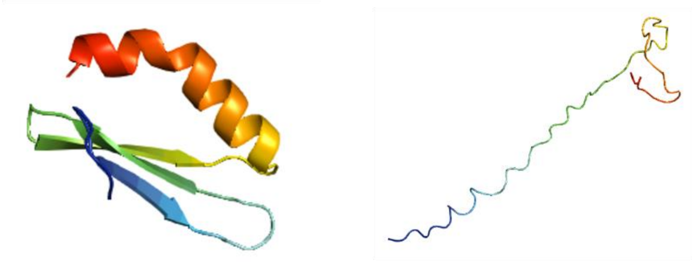

j) PstPEC6

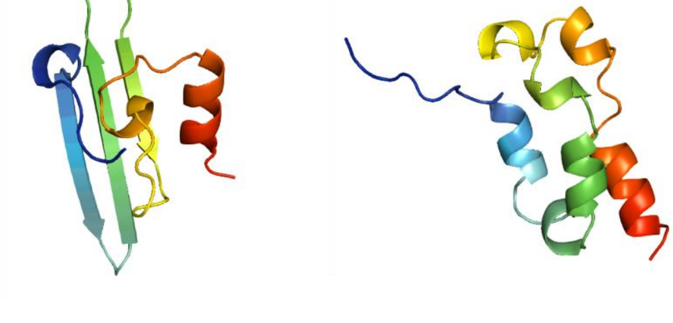

Supplement: Supplementary file 1 [file ijms-24-06262-s001.zip › ijms-2217391-supplementary.pdf]
